# Soil-based nano-graphene oxide and foliar selenium and nano-Fe influence physiological responses of 'Sultana' grape under salinity

**DOI:** 10.1038/s41598-022-08251-8

**Published:** 2022-03-10

**Authors:** Mohammad Ali Aazami, Lamia Vojodi Mehrabani, Tahereh Hashemi, Mohammad Bagher Hassanpouraghdam, Farzad Rasouli

**Affiliations:** 1 Department of Horticultural Science, Faculty of Agriculture, University of Maragheh, Maragheh, Iran; 2 Department of Agronomy and Plant Breeding, Azarbaijan Shahid Madani University, Tabriz, Iran

**Keywords:** Physiology, Plant sciences

## Abstract

Salinity is a worldwide stressor that influences the growth and productivity of plants. Some novel compounds like; graphene oxide and nutrients such as Se and Fe especially as nano form may improve plant responses to the environmental stress factors. The soil-based graphene oxide (0, 50, and 100 g kg^−1^) and the foliar applications of Se and nano-Fe (control and 3 mg L^−1^) were assayed on grapevine cv. Sultana under salinity (0, 50, and 100 mM NaCl). The top flavonoids, chlorophyll b, and plant dry weight belonged to graphene oxide and nano-Fe applications. CAT activity was improved in response to Se, nano-Fe, and graphene oxide (50 g kg^−1^). The least Fe, K, Se, N, Mg, Mn, and Zn content was recorded for 100 mM NaCl. In contrast, the higher data for K, Se, Ca, Mg, Zn and Mn were acquired with graphene oxide × foliar treatments. In general, graphene oxide treatment (50 g kg^−1^) × nano-Fe and Se foliar use ameliorated the adverse salinity effects with the improved biochemical and physiological responses of Sultana grape.

## Introduction

Salinity is one of the significant limitations of plant growth and causes considerable yield and economic losses for the plant producers^1^. High salinity damages plants via nutrient imbalances, impaired metabolism, ROS generation, growth inhibition, osmotic stress, and the reduced quality and productivity^1,2^. Under salinity, plants produce reactive oxygen species (ROS) such as singlet oxygen and superoxide anions in chloroplasts and mitochondria, which are toxic to lipids, proteins, carbohydrates, and DNA^1^. Salinity diminishes the ability of plants to absorb nutrients and increases the intracellular ion concentration, ultimately resulting in a severe reduction in the growth and development of plants^3^. Meanwhile, plants with the dynamic antioxidant enzymes pool can quieten damages caused by the environmental stress factors^4,5^.

Nowadays, nanotechnology is an inevitable part of human life. Furthermore, recent progress in nanotechnology has replaced traditional agricultural methods with modern production procedures^6^. During the past decade, nanotechnology has been extensively employed to alleviate the adverse effects of environmental stressors in plants production systems^7^. Nano-materials are molecules with 1–100 nm in size; having a crucial role in plants' growth and productivity, especially under stressful conditions^6,7^. Nanomaterials as fertilizers lessen the nutrient loss and even reduce the fertilizers' input^8^. Nanomaterials are highly mobile and active, and due to their low-cost production; their application is hugely economical^6,8^. Graphene nano-particle is extensively used in diverse domains of basic science, energy, and medicine^6,9^. The optical, mechanical, and electrical properties of graphene oxide particles make them reliable candidates for various applications in many scientific disciplines^7^. Soil-based application of graphene oxide improved the physicochemical properties of the soils^9^. Furthermore, a study in *Silybum marianum* revealed that graphene-oxide use increased chlorophyll content and RWC as well as improved the growth potential and yield of plants^10^. Anjum et al.^11^ in their study on bean reported that the application of low concentrations of graphene had a positive effect on plant growth and physiological traits. Fe plays a chief role in chloroplast formation, photosynthesis, enzymatic activity (catalase and peroxidase), nitrogen fixation, and respiration in plants^12,13^. In *Moringa peregrine*^14^ and *Vitis vinifera*^15^, iron nano-particle application under salinity improved growth potential and yield. Selenium plays an important role in the activity of antioxidant enzymes (glutathione and peroxidase) and improves photosynthesis rate and potassium uptake^16^. With *Anethum graveolens*, selenium under saline conditions facilitated the growth and productivity of plant as well^17^.

*Vitis vinifera* is a perennial plant of the Vitaceae family^2^. Grapes, forming 12.1% of Iran’s orchards (300,000 hectares) and producing about 3.2 million tons in 2019 ranked the second after pistachio among Iran’s non-oil exports^18^. Salinity is the most critical abiotic stressor having a noticeable negative impact on the performance of vineyards in the arid and semi-arid regions of the world^19^. Water quality (in terms of salinity) has an important effect on berry characteristics, nutritional components, and the yield of vineyards^20,21^. Walker et al.^2^ reported that salinity caused a decline in stomatal conductance and nutrient availability, reducing the grapes' yield. Considering the economic importance of grapevine and its products in various industries and the appropriate climatic conditions in Iran for their cultivation; the purpose of this study was to evaluate the effect of Se and nano-Fe foliar use and also the soil-based application of graphene oxide on the growth and some physiological traits of grapevines under salinity conditions.

## Results

### Aerial parts dry weight

Triple interaction effects were significant (p ≤ 0.01) on aerial parts' dry weight. The highest data was obtained by graphene oxide and foliar treatment of nano-Fe without salinity stress. Salinity drastically reduced this trait (Table 1).

**Table 1:** Mean comparisons for the effect of soil-based graphene oxide and foliar application of selenium and nano-iron under salinity on the growth, physiological traits, and antioxidant enzymes activity of grape cv. sultana.

Treatment composition	Aerial parts dry weight (g/pot)	Flavonoids content (mg g^−1^ DWt)	Total phenolics content (mg g^−1^ DWt)	Proline content (µg g^−1^ FWt)	SOD activity (µµmg^−1^protein)	H_2_O_2_ content (µmol g^−1^ FW)	CAT activity (unit/mg protein min^−1^)	Chl b content (mg g^−1^ FWt)
T1	36 ± 0.47^g-i^	1 ± 0.04^j^	85.6 ± 1.51^f-h^	19.6 ± 1.18^q^	5.4 ± 0.38^j^	1.5 ± 0.05^k-m^	17 ± 0.47^i-k^	0.23 ± 0.020^kl^
T2	49.6 ± 0.98^ab^	2.6 ± 0.1^c-e^	134 ± 2.76^a^	28.3 ± 0.98^p^	7.1 ± 0.19^hi^	1.3 ± 0.04^n^	22 ± 0.64^c-e^	0.8 ± 0.04^ab^
T3	49.6 ± 0.27^ab^	3 ± 0.07^c^	111 ± 3.56^c-e^	34 ± 0.47^o^	6.9 ± 0.4^h-j^	1.36 ± 0.02^ mn^	25 ± 0.47^ab^	0.5 ± 0.06^e-h^
T4	47b ± 0.94^c^	1.9 ± 0.07^gh^	93 ± 2.05^fg^	39.6 ± 1.08^n^	6.4 ± 0.53^h-j^	1.8 ± 0.04^ij^	19 ± 0.08^g-i^	0.66 ± 0.02^b-d^
T5	51 ± 1.18^a^	3.9 ± 0.07^ab^	119 ± 4.71^b-d^	43.3 ± 0.72^m^	6.8 ± 0.27^h-j^	1.4 ± 0.02^i-n^	20 ± 0.21^c-h^	0.62 ± 0.03^c-e^
T6	38 ± 0.98^fg^	2.7 ± 0.04^cd^	104 ± 0.72^e^	47.6 ± 0.72^k^	7.4 ± 0.25^gi^	1.3 ± 0.02^ mn^	22 ± 0.4^d-f^	0.43 ± 0.02^g-j^
T7	46.3 ± 0.98^bc^	1.9 ± 0.05^gh^	81 ± 3.92^h^	53 ± 0.47^ij^	5.9 ± 0.04^ij^	1.6 ± 0.05^f-l^	19 ± 0.71^g-i^	0.5 ± 0.04^e-h^
T8	52.3 ± 0.27^a^	3.6 ± 0.12^b^	133 ± 2.88^a^	54.6 ± 0.27^i^	7.7 ± 0.24^f-h^	1.3 ± 0.07^mn^	22 ± 0.36^d-f^	0.9 ± 0.04^a^
T9	45.3 ± 0.98^cd^	2.9 ± 0.04^c^	108 ± 1.24^c^	63.2 ± 1.18^gh^	6.7 ± 0.24^h-j^	1.2 ± 0.04^n^	22 ± 0.4^d-f^	0.7 ± 0.02^bc^
T10	38.3 ± 0.72^fg^	1.9 ± 0.05^gh^	90.6 ± 0.27^f-h^	37 ± 3.09^no^	8 ± 0.38^f-h^	2.6 ± 0.07^d-f^	16 ± 0.47^k^	0.3 ± 0.03^j-l^
T11	42.3 ± 1.08^de^	2.4 ± 0.07^d-f^	113 ± 1.88^c-e^	44 ± 0.47^ lm^	8 ± 0.41^f-h^	1.9 ± 0.09^i^	19 ± 0.42^g-i^	0.7 ± 0.05^bc^
T12	40.6 ± 1.78^ef^	2 ± 0.02^gh^	120 ± 0.27^bc^	47 ± 0.47^kl^	7 ± 0.63^f-h^	1.5 ± 0.02^k-m^	19 ± 0.23^g-i^	0.7 ± 0.04^bc^
T13	42d ± 1.63^c^	2.2 ± 0.11^e-g^	92 ± 2.94^f-h^	65 ± 0.47^fg^	10 ± 0.07^c-e^	2.3 ± 0.08^h^	16 ± 0.25^k^	0.6 ± 0.04^c-f^
T14	40.3 ± 1.18^ef^	4.2 ± 0.11^a^	125 ± 1.9^ab^	68 ± 1.24^d-f^	10 ± 0.27^b-d^	1.8 ± 0.09^ih^	25 ± 0.45^ab^	0.7 ± 0.05^bc^
T15	40.6 ± 0.54^ef^	2 ± 0.08^c^	105 ± 2.35^e^	73 ± 0.47^c^	9.7 ± 0.1^c-e^	1.7 ± 0.07^i-k^	23 ± 0.44^b-d^	0.5 ± 0.01^e-h^
T16	34.6 ± 1.90^hi^	3.2 ± 0.09^c^	84.3 ± 4.28^f-h^	69.3 ± 0.54^de^	9.2 ± 0.43^d-e^	2.4 ± 0.05^gh^	19 ± 0.57^g-i^	0.3 ± 0.01^i-k^
T17	34.6 ± 0.27^hi^	2.7 ± 0.09^cd^	120 ± 1.65^bc^	67 ± 0.81^ef^	11.1 ± 0.05^bc^	1.9 ± 0.07^i^	24 ± 0.71^a-d^	0.46 ± 0.02^f-i^
T18	35.3 ± 0.72^g-i^	2.2 ± 0.04^fg^	112.3 ± 1.18^c-e^	68.3 ± 0.54^d-f^	12.2 ± 0.09^ab^	1.8 ± 0.07^ij^	26 ± 0.73^a^	0.3 ± 0.04^j-l^
T19	29 ± 0.54^k^	1.5 ± 0.25^ij^	70.3 ± 1.9^i^	50 ± 0.47^jk^	10.2 ± 0.3^c-e^	3.8 ± 0.09^a^	18.8 ± 2.03^h-j^	0.4 ± 0.01^g-j^
T20	30 ± 0.72^jk^	2 ± 0.12^gh^	89 ± 1.18^f-h^	60.3 ± 0.72^h^	9.7 ± 0.59^c-e^	3 ± 0.02^c^	18 ± 0.68^h-j^	0.3 ± 0.02^i-k^
T21	36 ± 0.81^g-i^	1.7 ± 0.12^hi^	89 ± 4.28^f-h^	55 ± 0.72^i^	13.1 ± 0.56^a^	2.8 ± 0.02^cd^	21 ± 1.18^d-g^	0.5 ± 0.02^d-g^
T22	33 ± 0.47^h-j^	1.7 ± 0.10^hi^	82 ± 3.03^gh^	69 ± 0.47^de^	10.1 ± 0.55^c-e^	3.5 ± 0.07^b^	22 ± 0.72^c-e^	0.6 ± 0.02^c-e^
T23	34.6 ± 1.08^hi^	1.9 ± 0.22^gh^	109 ± 4.83^de^	71 ± 2.05^cd^	11.1 ± 0.79^bc^	2.7 ± 0.02^de^	23 ± 0.98^b-d^	0.36 ± 0.05^h-k^
T24	34.3 ± 0.72^hi^	2 ± 0.05^gh^	81 ± 1.96^h^	73.6 ± 0.27^c^	10.9 ± 0.59^bc^	3 ± 0.04^c^	25 ± 0.72^ab^	0.4 ± 0.05^g-j^
T25	29 ± 0.47^k^	2 ± 0.09^gh^	81 ± 2.59^h^	79.3 ± 0.27^b^	8.9 ± 0.17^e-g^	3.4 ± 0.08^b^	19.2 ± 0.19^g-i^	0.16 ± 0.01^l^
T26	33.6 ± 0.54^h-j^	1.9 ± 0.04^gh^	93 ± 2.37^f^	82.3 ± 0.72^ab^	10.5 ± 0.43^ cd^	2.53 ± 0.07^e-g^	20 ± 0.32^e-h^	0.5 ± 0.03^e-h^
T27	32.6 ± 0.72^ij^	2.7 ± 0.15^cd^	94 ± 1.24^f^	84 ± 0.47^a^	10.7 ± 0.47^b-d^	2.5 ± 0.04^f-h^	23 ± 0.29^b-e^	0.5 ± 0.03^d-g^
**Significance**								
NaCl	**	**	**	**	**	**	ns	**
Graphene	*	**	*	**	**	**	**	*
NaCl × graphene	**	**	**	**	**	**	**	**
Spray	**	**	**	**	**	**	**	**
NaCl × spray	**	**	**	**	ns	**	**	**
Graphene × spray	**	**	**	**	**	ns	ns	**
NaCl × graphene × spray	**	**	**	*	**	**	**	**

Similar letters show no meaningful difference at 5% probability level by Duncan’s Multiple Range Test. Data are mean ± SD (n = 3 replicates). ns, ** and *: Non-significant, significant at 1 and 5 percentage probability levels, respectively.

T1: NaCl_0_ × graphene_0_ × Spray_0_; T10: NaCl_50_ × graphene_0_ × Spray_0_; T19: NaCl_100_ × graphene_0_ × Spray_0_.

T2: NaCl_0_ × graphene_0_ × Spray_Fe_; T11: NaCl_50_ × graphene_0_ × Spray_Fe_; T20: NaCl_100_ × graphene_0_ × Spray_Fe_.

T3: NaCl_0_ × graphene_0_ × Spray_Se_; T12: NaCl_50_ × graphene_0_ × Spray_Se_; T21: NaCl_100_ × graphene_0_ × Spray_Se_.

T4: NaCl_0_ × graphene_50_ × Spray_0_; T13: NaCl_50_ × graphene_50_ × Spray_0_; T22: NaCl_100_ × graphene_50_ × Spray_0_.

T5: NaCl_0_ × graphene_50_ × Spray_Fe_; T14: NaCl_50_ × graphene_50_ × Spray_Fe_; T23: NaCl_100_ × graphene_50_ × Spray_Fe_.

T6: NaCl_0_ × graphene_50_ × Spray_Se_; T15: NaCl_50_ × graphene_50_ × Spray_Se_; T24: NaCl_100_ × graphene_50_ × Spray_Se_.

T7: NaCl_0_ × graphene_100_ × Spray_0_; T16: NaCl_50_ × graphene_100_ × Spray_0_; T25: NaCl_100_ × graphene_100_ × Spray_0_.

T8: NaCl_0_ × graphene_100_ × Spray_Fe_; T17: NaCl_50_ × graphene_100_ × Spray_Fe_; T26: NaCl_100_ × graphene_100_ × Spray_Fe_.

T9: NaCl_0_ × graphene_100_ × Spray_Se_; T18: NaCl_50_ × graphene_100_ × Spray_Se_; T27: NaCl_100_ × graphene_100_ × Spray_Se_.

### Total phenolics and flavonoids content

These traits were affected by the triple interaction effects as well (p ≤ 0.01). The top total flavonoids content belonged to foliar application of nano-Fe with 50 g kg^−1^ graphene oxide under control and moderate salinity stress conditions (76% more than control). Moreover, the highest phenolics content was recorded for no-saline conditions with nano-Fe application. Graphene oxide × nano-Fe application improved phenolics content under 50 mM NaCl salinity (Table 1).

### Malondialdehyde and hydrogen peroxide content

**Figure 1:**
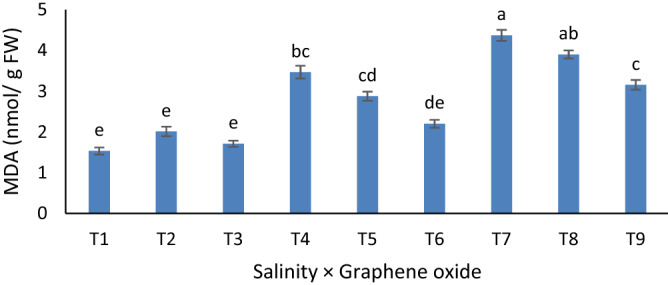
Mean comparison for the interactions of salinity stress and soil-based application of graphene oxide on malondialdehyde content in grapevine. Similar letters show no meaningful difference at 5% probability level by Duncan’s Multiple Range Test. Data are mean ± SD (n = 3 replicates). T1: NaCl_0_ × graphene_0_, T2: NaCl_0_ × graphene_50_, T3: NaCl_0_ × graphene_100_, T4: NaCl_50_ × graphene_0_, T5: NaCl_50_ × graphene_50_, T6: NaCl_50_ × graphene_100_, T7: NaCl_100_ × graphene_0_, T8: NaCl_100_ × graphene_50_, T9: NaCl_100_ × graphene_100_.

H_2_O_2_ content was influenced by the triple interaction effects of treatments as well (p ≤ 0.01). The highest content of H_2_O_2_ (3.8 µmol g^−1^ Fwt) was recorded for 100 mM salinity alone. H_2_O_2_ content declined with graphene oxide and Se. With salinity adding up, MDA content was correspondingly increased. Control plants attained the most minor MDA content (Fig. 1). Foliar treatment of nano-Fe and Se do not significantly influenced these traits.

### Catalase and superoxide dismutase activity

CAT and SOD activities were also responsive to the triple interaction effects (p ≤ 0.01). The maximum of CAT activity was obtained with control, 50 and 100 mM NaCl with Se foliar spray. SOD activity was increased under salinity and Se treatment (41% more than control).

### Proline and total soluble solids content

Treatment effects were not significant for TSS, but, they were significant for proline content (p ≤ 0.05). The lowest proline content (19.6 µg g^−1^ Fwt) was recorded for control and, the highest recorded data (84 µg g^−1^ Fwt) belonged to 100 mM NaCl × Se × 100 g kg^−1^ graphene oxide (Table 1). Graphene oxide utilization positively impacted TSS content under control and saline conditions.

### Chlorophyll’s content

**Figure 2:**
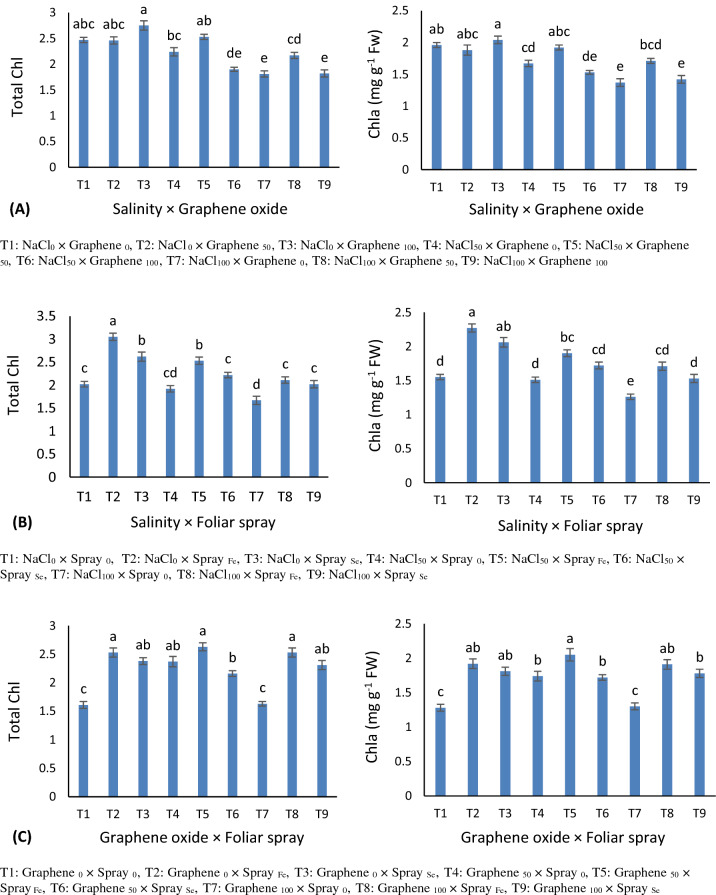
Mean comparisons for the interactions of salinity and soil-based application of graphene oxide (**A**), salinity and foliar spray (**B**), graphene oxide, and foliar spray (**C**) on total chlorophyll and chlorophyll a content in grapevine. Similar letters show no meaningful difference at 5% probability level by Duncan’s Multiple Range Test. Data are mean ± SD (n = 3 replicates).

Triple interaction effects were significant (p ≤ 0.01) just for chlorophyll b content. The highest amount of chlorophyll b was obtained by nano-Fe. Under salinity, graphene oxide and nano-Fe improved chl b content (Table 1). Graphene oxide application under normal and saline conditions increased chl a and total chlorophylls content. Foliar spray of nano-Fe and Se partially increased chl a and total chlorophyll content under salinity. However, the highest amounts of total chl and chl a belonged to graphene oxide of 50 g kg^−1^ × nano-Fe applications (Fig. 2).

### Elemental content

Tripartite effects were significant (p ≤ 0.01) for K, Mg, Mn, Na, Zn, Ca, N, Se and Fe. The greatest amount of K (51 g kg^−1^ Dwt) belonged to control × 50 g kg^−1^ graphene oxide × Se (37% more than control). Fe and Se contents were correspondingly responded to the foliar spray treatments. Graphene oxide and nano-Fe improved N content of plants. The least N content (7 g kg^−1^ Dwt) belonged to 100 mM salinity. Graphene oxide and Se treatment under control and mild salinity conditions improved the tissues' Ca and Mg content. Mn attained its highest amount with control and 50 mM salinity × graphene oxide × Se. 50 g kg^−1^ graphene oxide and Se with control plants had the top Zn content. In contrast, the least Zn content belonged to 100 mM salinity. Salinity linearly increased Na content. However, Se and nano-Fe treatment under salinity reduced Na content of plants (Table 2).

**Table 2:** Mean comparisons for the effects of soil-based graphene oxide and foliar application with magnetic nano-iron and selenium under salinity on the elemental content of grapevine cv. Sultana.

Treatment composition	K (g kg^-1^ Dwt)	Na (g kg^−1^ Dwt)	Fe (mg kg^−1^ Dwt)	Se (mg kg^−1^ Dwt)	N (g kg^−1^ Dwt)	Ca (g kg^−1^ Dwt)	Mg (g kg^−1^ Dwt)	Zn (mg kg^−1^ Dwt)	Mn (mg kg^−1^ Dwt)
T1	32 ± 0.7^i-k^	3.3 ± 0.47^h^	153.4 ± 2.55^kl^	34 ± 1.24^gh^	12 ± 0.47^f-i^	18 ± 0.19^g-l^	2.1 ± 0.04^d^	21 ± 0.27^f-l^	21 ± 0.42^ef^
T2	43 ± 0.47^c-e^	3.6 ± 0.87^h^	372.3 ± 9.27^a^	39 ± 1.18^g^	18.6 ± 0.72^b^	19 ± 0.47^f-k^	2 ± 0.04^d-f^	25 ± 0.64^de^	20 ± 0.57^e-g^
T3	45 ± 0.38^bc^	3.3 ± 0.25^h^	204 ± 2.86^f^	97 ± 2.59^b^	15 ± 0.47^de^	22 ± 0.34^dc^	2.5 ± 0.07^ab^	27 ± 0.89^b-d^	25 ± 0.81^bc^
T4	46 ± 0.81^bc^	12 ± 0.48^cd^	179 ± 6.33^h-j^	34 ± 1.41^gh^	19 ± 0.72^ab^	19 ± 0.3^d-i^	2.3 ± 0.02^c^	29 ± 0.37^ab^	27 ± 0.35^a^
T5	43 ± 0.94^c-e^	9 ± 0.39^eg^	318 ± 8.14^b^	33 ± 0.81^g-i^	21.9 ± 0.98^a^	19 ± 0.51^d-i^	1.9 ± 0.07^d-g^	22 ± 0.77^f-g^	20 ± 0.72^e-g^
T6	51 ± 0.27^a^	7.6 ± 0.28^eg^	272 ± 7.2^ cd^	96 ± 4.25^b^	16 ± 0.47^ cd^	26 ± 0.94^a^	2.7 ± 0.08^a^	31 ± 1.2^a^	28 ± 0.89^a^
T7	42 ± 0.72^c-e^	14.3 ± 1.1^b^	160 ± 3.6^j-i^	31.6 ± 0.72^h-j^	15 ± 0.72^ cd^	20 ± 0.25^c-g^	2.03 ± 0.05^de^	23 ± 0.51^ef^	25 ± 0.5^bc^
T8	40 ± 0.98^e-f^	14.8 ± 0.39^b^	312 ± 9.75^b^	30 ± 0.81^h-j^	17 ± 0.72^bc^	17 ± 0.54^il^	1.8 ± 0.04^f-i^	19 ± 0.92^h-k^	21 ± 0.94^ef^
T9	45 ± 1.41^bc^	11.4 ± 1.2^de^	277 ± 4.03^c^	105 ± 2.99^a^	14.6 ± 0.27^de^	25 ± 0.96^a^	2.4 ± 0.02^bc^	28 ± 1.11^bc^	27 ± 0.33^a^
T10	29 ± 1.24^kl^	14.1 ± 0.57^i^	141 ± 2.99^i^	84.3 ± 0.27^bc^	8.3 ± 0.54^kl^	18 ± 0.66^g-l^	1.56 ± 0.05^jk^	20 ± 0.47^g-j^	20 ± 0.54^e-g^
T11	31 ± 1.36^i-k^	8.8 ± 0.38^fg^	277 ± 8.73^c^	26 ± 0.27^h-j^	11 ± 0.47^g-j^	17 ± 0.72^j-l^	1.5 ± 0.02^ k^	20 ± 0.72^h-k^	18 ± 0.47^h-j^
T12	31 ± 1.24^i-k^	9 ± 0.28^fg^	201 ± 1.51^fg^	63 ± 1.44^d^	12 ± 0.47^f-i^	22 ± 0.95^bc^	1.9 ± 0.05^d-g^	25 ± 0.74^de^	25 ± 0.49^bc^
T13	44 ± 0.3^d-f^	12.3 ± 0.38^i^	177 ± 4.48^h-j^	29 ± 0.27^h-j^	10 ± 0.47^ik^	21 ± 0.29^b-e^	2 ± 0.04^d-f^	27 ± 0.23^b-d^	23 ± 0.27^b-d^
T14	45 ± 0.47^b-d^	3.3 ± 0.54^h^	256 ± 5.44^de^	29 ± 0.94^h-j^	17 ± 0.72^bc^	21 ± 0.73^b-d^	1.8 ± 0.05^e-h^	21 ± 0.66^f-h^	20 ± 0.24^e-g^
T15	48 ± 2.4^bc^	3.3 ± 0.21^h^	204 ± 2.16^f^	80 ± 6.38^c^	13 ± 0.47^e-g^	26 ± 0.54^a^	2 ± 0.04^d-f^	29 ± 0.38^ab^	25 ± 0.66^b^
T16	33 ± 1.96^h-j^	10.2 ± 1.^1d-f^	168 ± 4.89^i-k^	27 ± 0.47^h-j^	12 ± 0.72^f-h^	22 ± 0.53^bc^	1.9 ± 0.02^d-g^	25 ± 0.47^de^	25 ± 0.72^b^
T17	30 ± 0.27^jk^	9.1 ± 0.7^eg^	239 ± 4.71^e^	28 ± 0.72^h-j^	16 ± 0.47^cd^	17.1 ± 0.54^kl^	1.6 ± 0.05^i-k^	20 ± 0.81^g-j^	19 ± 0.76^f-i^
T18	37 ± 1.24^gh^	9.7 ± 0.3^eg^	203±3.21^f^	74.3 ± 1.65^c^	13 ± 0.72^ef^	20.2 ± 0.32^c-h^	1.9 ± 0.02^d-g^	29 ± 0.13^ab^	28 ± 0.81^a^
T19	25 ± 0.72^ m^	17.3 ± 0.6^ab^	120 ± 0.81^m^	23.6 ± 1.65^j^	7 ± 0.47^ l^	14.3 ± 0.25^ m^	1.1 ± 0.02^ l^	18 ± 0.62^jk^	18 ± 0.19^g-j^
T20	25 ± 0.27^ lm^	14 ± 0.7^bc^	208 ± 5.22^f^	25 ± 0.47^ij^	10 ± 0.72^h-k^	13.5 ± 0.19^m^	1.1 ± 0.07^l^	17 ± 0.52^k^	15 ± 0.6^k^
T21	38 ± 0.32^fg^	13 ± 0.48^bc^	197 ± 3.66^f-h^	55 ± 1.9^e^	9.6 ± 0.27^jk^	17.6 ± 0.72^i-l^	1.56 ± 0.02^jk^	25 ± 0.47^de^	23 ± 0.36^cd^
T22	34 ± 0.44^g-i^	14 ± 0.12^i^	145 ± 6.8^i^	27 ± 0.72^h-j^	11 ± 0.47^g-j^	20 ± 0.16^c-f^	1.2 ± 0.11^ l^	22 ± 0.81^f-h^	21 ± 0.48^de^
T23	31 ± 0.79^jk^	10.2 ± 0.1^d-f^	218 ± 4.5^f^	33 ± 0.98^gh^	12 ± 0.47^f-i^	19 ± 0.25^e-j^	1.7 ± 0.02^g-j^	21 ± 0.47^g-j^	17 ± 0.5^i-j^
T24	38 ± 0.72^fg^	10.9 ± 0.2^d-f^	182 ± 2.68^g-i^	59 ± 2.12^de^	9.3 ± 0.27^j-k^	22 ± 0.47^b^	1.9 ± 0.04^d-h^	26 ± 0.94^c-e^	23 ± 0.28^b-d^
T25	29 ± 0.27^k^	9.7 ± 0.41^eg^	150 ± 2.32^kl^	25 ± 1.88^ij^	8.3 ± 0.27^kl^	18 ± 0.47^h-l^	1.7 ± 0.04^h-k^	19.6 ± 0.54^g-k^	19 ± 0.3^f-h^
T26	31 ± 0.62^ik^	8.8 ± 0.31^fg^	238 ± 8.06^e^	23.6 ± 1.78^j^	12.3 ± 0.27^f-h^	16 ± 0.68^ l^	1.5 ± 0.09^ k^	18.7 ± 0.3^l-k^	16 ± 0.07^j-k^
T27	33 ± 0.27^h-i^	9 ± 0.27^fg^	206 ± 4.48^f^	46.6 ± 1.18^f^	13 ± 0.47^e-g^	19 ± 0.47^d-h^	1.8 ± 0.07^e-h^	28.7 ± 0.21^ab^	20 ± 0.45^e-g^
**Significance**									
NaCl	**	**	**	**	**	**	**	**	**
Graphene	**	**	**	**	**	**	**	**	**
NaCl × graphene	**	**	**	**	**	**	**	**	**
Spray	**	**	**	**	**	**	**	**	**
NaCl × spray	ns	ns	**	*	**	*	**	*	**
Graphene × spray	*	ns	**	ns	**	**	**	**	**
NaCl × graphene × spray	**	**	**	**	**	**	**	**	**

Similar letters show no meaningful difference at 5% probability level by Duncan’s Multiple Range Test. Data are mean ± SD (n = 3 replicates). ns, ** and *: non-significant, significant at 1 and 5 percentage probability levels, respectively.

T1: NaCl_0_ × graphene_0_ × Spray_0_; T10: NaCl_50_ × graphene_0_ × Spray_0_; T19: NaCl_100_ × graphene_0_ × Spray_0_.

T2: NaCl_0_ × graphene_0_ × Spray_Fe_; T11: NaCl_50_ × graphene_0_ × Spray_Fe_; T20: NaCl_100_ × graphene_0_ × Spray_Fe_.

T3: NaCl_0_ × graphene_0_ × Spray_Se_; T12w: NaCl_50_ × graphene_0_ × Spray_Se_; T21: NaCl_100_ × graphene_0_ × Spray_Se_.

T4: NaCl_0_ × graphene_50_ × Spray_0_; T13: NaCl_50_ × graphene_50_ × Spray_0_; T22: NaCl_100_ × graphene_50_ × Spray_0_.

T5: NaCl_0_ × graphene_50_ × Spray_Fe_; T14: NaCl_50_ × graphene_50_ × Spray_Fe_; T23: NaCl_100_ × graphene_50_ × Spray_Fe_.

T6: NaCl_0_ × graphene_50_ × Spray_Se_; T15: NaCl_50_ × graphene_50_ × Spray_Se_; T24: NaCl_100_ × graphene_50_ × Spray_Se_.

T7: NaCl_0_ × graphene_100_ × Spray_0_; T16: NaCl_50_ × graphene_100_ × Spray_0_; T25: NaCl_100_ × graphene_100_ × Spray_0_.

T8: NaCl_0_ × graphene_100_ × Spray_Fe_; T17: NaCl_50_ × graphene_100_ × Spray_Fe_; T26: NaCl_100_ × ghraphene_100_ × Spray_Fe_.

T9: NaCl_0_ × graphene_100_ × Spray_Se_; T18: NaCl_50_ × graphene_100_ × Spray_Se_; T27: NaCl_100_ × graphene_100_ × Spray_Se_.

**Figure 3:**
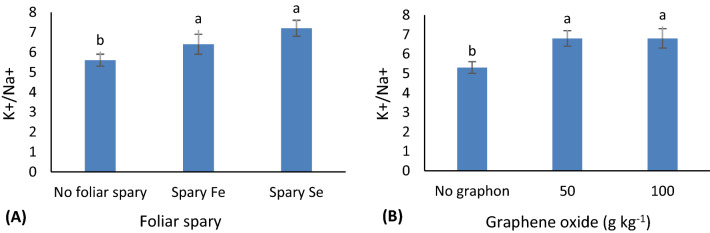
Mean comparisons for the effects of foliar spray of Fe and Se (**A**) and graphene oxide soil-based application (**B**) on K^+^/Na^+^ in grapevine cv. Sultana. Similar letters show no meaningful difference at 5% probability level by Duncan’s Multiple Range Test. Data are mean ± SD (n = 3 replicates).

**Figure 4:**
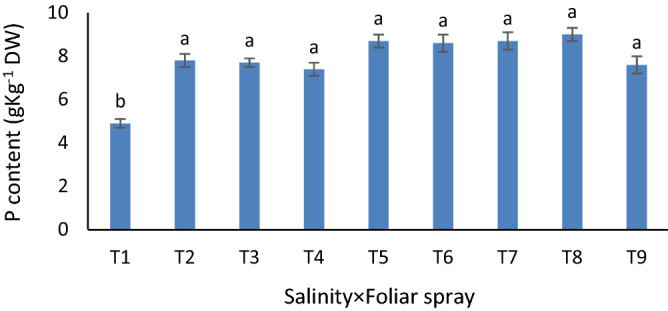
Mean comparisons for the effect of soil-based application of graphene oxide and foliar application with magnetic nano-iron and selenium under salinity on P content of grapevine cv. Sultana. Similar letters show no meaningful difference at 5% probability level by Duncan’s Multiple Range Test. Data are mean ± SD (n = 3 replicates). T1: NaCl_0_ × Spray_0_, T2: NaCl_0_ × Spray_Fe_, T3: NaCl_0_ × Spray_Se_, T4: NaCl_50_ × Spray_0_, T5: NaCl_50_ × Spray_Fe_, T6: NaCl_50_ × Spray_Se_, T7: NaCl_100_ × Spray_0_, T8: NaCl_100_ × Spray_Fe_, T9: NaCl_100_ × Spray_Se_.

For K^+^/Na^+^ ratio, the individual effects were significant. Foliar treatment vs. no-foliar treatment and graphene oxide application vs. its non-use; increased K^+^/Na^+^ ratio (Fig. 3). P content was responsive to Se and nano-Fe application under both control and saline conditions (Fig. 4).

### Correlation coefficient

**Figure 5:**
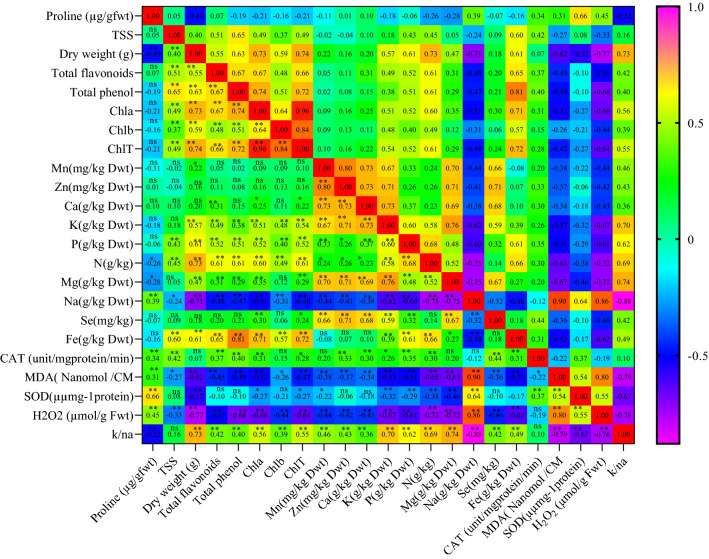
Pearson correlations among evaluated traits of grapevine cv. Sultana under salinity and treated by Se and nano Fe. **, * and ^ns^ correlation were significant at 1% and 5% of probability level and non-significant, respectively. TSS (total soluble solids), Chl a (chlorophyll a), Chl b (chlorophyll b), Chl T (total chlorophyll), CAT (catalase activity), SOD (superoxide dismutase activity), MDA (malondialdehyde content).

The results (Fig. 5) revealed a significant positive correlation between the activity of SOD and proline content. Furthermore, there was a positive relationship between chl a, N content, K^+^/Na^+^ ratio, and plant dry weight. A positive correlation was traced between Fe and total phenolics content. The same trend was traced between chl a and total phenolics. Contrarily, N content negatively correlated with H_2_O_2_ and Na^+^. A positive correlation was recorded between Na, K^+^/Na^+^ ratio, and H_2_O_2_.

Selenium content was positively related to K and Zn content. Instead, a reverse relationship was recorded between H_2_O_2_ content, N, Mg, and plant dry weight. There was a direct correlation between H_2_O_2_, Na content, and MDA. Alike correlation analysis, the loading plot of the attributes revealed that the correlated characteristics were arranged at a close distance on the plot. For instance, traits such as proline content and SOD activity were positively correlated and placed on the left side of the plot.

**Figure 6:**
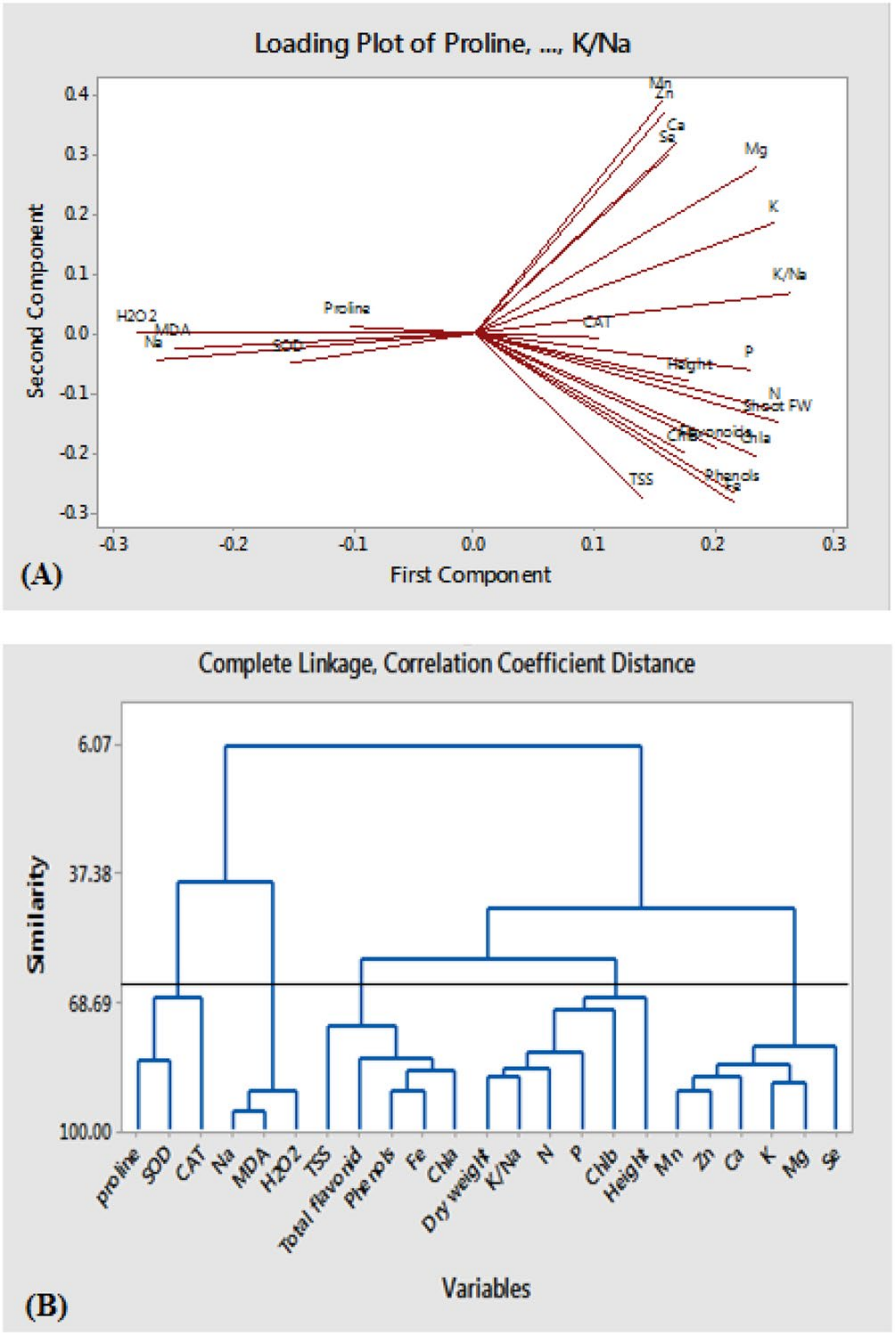
The loading plot for the evaluated traits of grapevine cv. Sultana under salinity and treated by Se and nano-Fe included in principal component analysis. The studied traits included TSS (total soluble solids), Chl a (chlorophyll a), Chl b (chlorophyll b), Chl T (total chlorophyll), CAT (catalase activity), SOD (superoxide dismutase activity), MDA (malondialdehyde content) (**A**). The cluster dendrogram is based on the Ward method among evaluated traits of grapevine cv. Sultana under salinity and treated by Se and nano-Fe. The studied traits included TSS (total soluble solids), Chl a (chlorophyll a), Chl b (chlorophyll b), Chl T (total chlorophyll), CAT (catalase activity), SOD (superoxide dismutase activity), MDA (malondialdehyde content) (**B**).

Meanwhile, most of the traits were placed on the right side (Fig. 6A). Based on the cluster analysis; the evaluated traits were grouped similarly to the biplot and correlation analysis, where, 5 groups were characterized. Proline, SOD and CAT; Na, MDA and H_2_O_2_; TSS, flavonoids, phenolics, Fe and chl a; and, Mn, Zn, Ca, K, Mg and Se (Fig. 6B).

## Discussion

Salinity harmed the plants growth, physiological and biochemical responses; but, foliar application with Se and nano-Fe and the soil-based application of graphene oxide improved the physiological responses. The use of Se under salinity stress increased *Vitis* dry weight^22^ due to improved root growth system and the enhanced nutrients uptake^23^. Using nano-fertilizers is a crucial step toward sustainable agricultural production systems. Nano-fertilizers improve nutrient use efficiency and even diminish the ecosystems' pollution by the low fertilizers input^6^. Pandy et al.^8^ reported that graphene oxide under NaCl stress reduces sodium uptake and increases the expression of aquaporins in the plant. Under salinity, graphene oxide had a positive role in photosynthesis, soluble sugar content, and cell membrane stability^8,10^. Fe plays a prominent role in photosynthesis and increases plants' yield. Due to the role of Fe in the biosynthesis of cytochromes and chlorophylls, its highest concentration is observed in photosynthetic cells^24^. Under salinity, Se treatment ameliorated the activity of MAPK7 and MAPK5 (protein kinases) and calcium-dependent protein kinase (CPK11) by regulating the expression of the related genes^25^.

Chrysargyris et al.^26^ reported a positive correlation between the total phenolics content of the plant and its antioxidant properties. Selenium plays a functional role in improving plant growth. Se seems to increase the expression of *UFGT* and *F3H* genes (involved in anthocyanin metabolism) in lettuce^27^. It was also found that under salinity and Se application, the activity of PAL increased, which changes the direction of secondary metabolites towards the production of phenolic compounds^28^. The results of a study on grapes showed that the use of Se under stress conditions increased Fe and flavonoids content^22^.

The high H_2_O_2_ content under salinity was reported in rice^29^. Furthermore, in grapevine, salinity increased H_2_O_2_ content, but, nano-Fe treatment considerably reduced H_2_O_2_ amount^15^. Under stress conditions, molecular oxygen acts as an electron acceptor, resulting in ROS radicals such as single oxygen, hydroxyl, superoxide, and hydrogen peroxide. These compounds are highly oxidizing and can cause cell membrane destruction^29^. Hydrogen peroxide plays a role in reducing stress's side-effects (conversion of H_2_O_2_ into water) via scavenging free radicals by activating ascorbate and glutathione reductase enzymes^30^.

The results of a study on tomato^31^ and garlic^28^ showed that Se application under salinity increased catalase and SOD activity. SOD and catalase activity in grapevine was intensified in nano-Fe treated plants under salinity conditions^15^. Studies showed that increasing the activity of antioxidant enzymes such as catalase and SOD in plants during stress; plays substantial action in removing free radicals produced in the cell. Catalase acts in removing hydrogen peroxide and thus helps the plant survive under stress^26^. Selenium seems to play a role in the activity of antioxidant enzymes and osmotic regulation in the plant and thus protects cells against stressors^17^.

A study on grape^22^ showed that Se treatment under salinity increased their proline content. A study performed on mint found that the use of Fe nanoparticles increased plant’s proline content under stressful conditions^32^. The role of proline in reducing the negative effects of salinity stress (eliminating oxygen free radicals, reducing cell acidification, regulating osmotic potential, and stability of cell membranes) was proven in several other plants^22,32,33^. Studies by Hare et al.^34^ on Arabidopsis revealed that 1PDH is expressed at the cellular level and reduces the effects of stress on the plant by increasing proline production. Under short-term stress conditions, proline acts as a carbon, nitrogen, and energy source for the cell. Salinity stress reduces cell membrane integrity. The use of graphene oxide under salinity reduced MDA generation^35^. The optimized graphene oxide concentration is essential for plant growth mainly due to the impact on cell proliferation and cell growth^36^.

Furthermore, the suitable graphene oxide concentration is crucial for plant growth due to the generation of hydrogen peroxide and the prevention of cell wall proteins and lipids peroxidation^11^. In grapevine, selenium under salinity conditions increased the pigment content in plant^31^. Selenium has prominent roles in chlorophyll content and by the function on the maintenance of chlorophylls structure; plays a dominant role in reducing salinity side-effects. Seemingly, the tiny size of nano-Fe fertilizers enhances their absorption and translocation and thereby smoothens the salinity depression^24^. Under saline conditions, Ca^2+^, K^+^, and Fe concentrations greatly decline in plants, leading to the reduced photosynthesis potential mainly via the least stomatal conductance and the declined CO_2_ acquisition^37,38^. In *Silybum marianum*, under salinity and with graphene oxide application, TSS content was increased^10^. Under stressful conditions, cell water content declines, and the cells are emergently producing low molecular weight compounds to combat the situation. Therefore, with stressful conditions, the use of graphene oxide is essential for fulfilling cell requirements by the increase in sugars biosynthesis. By regulating cell osmotic potential, sugars play a pivotal role in protecting enzymes against inorganic ions, mainly Na^+^, in between cell spaces. Moreover, sugars are the primary energy sources for the cells^9–11^.

High sodium and chlorine availability under saline environments lead to imbalances in the absorption of nutrients by the plant, hindering the growth potential and yield^39^. Due to the high similarity between Na^+^ and K^+^ ions; potassium absorption is interrupted by the Na^+^ high availability. Meanwhile, the high K^+^ content of plants devotes reliable salt tolerance properties. The disintegration of cell membranes due to salinity stress causes non-selective absorption of sodium ions in plants, leading to the accumulation of salts in the aerial parts of the plant and causing serious damage. Sodium accumulation in the aerial part tissues reduces calcium and potassium content due to the antagonistic behavior between sodium, potassium, and calcium. Similar results were reported for the high sodium content under salinity stress conditions in *Moringa peregrine*^14,15,25^. Potassium is an essential nutrient that plays a chief role in water uptake and transport, cell development, osmotic regulation, and the stomata's behaviour^1,40^. Given the multiple roles of potassium in the cells, potassium action in osmotic regulation is less costly than the supply of other organic compounds^41^. The obstacles in potassium uptake under salinity are mainly due to the antagonistic competition between sodium and potassium^1^. It seems that the high copy numbers of *OSNHX1* gene in rice influenced the potassium to sodium ratio under stress conditions by the sodium selenite treatment and increased the plant stress tolerance^29^. Mozafari et al.^15^ reported similar results regarding the increase in potassium content of plants due to Se spraying under saline conditions. The increased sodium uptake causes ion toxicity and ionic imbalances in the cell, with huge adverse effects on potassium uptake. The results of a study conducted by Almeida et al.^42^ showed that Se use under stress conditions hastened the activity of OSNHX1 as a Na^+^/H ^+^ antiport. Otherwise, the stimulated water transfer to the aerial parts helps the plant to withstand stress conditions. The diminished Fe absorption under salinity goes to ion imbalances and disorders in the plant growth and performance. Fe is a vital nutrient that plays eminent functions in chloroplast development, chlorophyll biosynthesis, thylakoids formation, and DNA and RNA biosynthesis^43^. Seemingly, the effect of Fe nanoparticles on plant growth is related to their size and high specific surface area^44^. The increase in P content by the foliar application of nano-Fe has been reported in peppermint as well^32^. The increase in phosphorous content by the salinity may be due to the loss of plants control on P absorption and translocation toward the aerial parts^45^. El-Fouly et al.^46^ noted that salinity reduced P uptake and amino acids biosynthesis in the wheat plant. In saline soils, the absorption of H_2_PO_4_ ions declines mainly due to the competition with Cl^-^, which drastically impacts plant growth and yield. P has great action in photosynthesis potential, and with the salinity depression, photosynthesis drastically declines^1^. In grapevine under salinity, Na^+^ content of aerial parts increased but the tissue K^+^ content declined^15^. Na^+^ accumulation interferes with the osmotic balance and membrane integrity. Furthermore, with Na^+^ high concentration in the nutrient solution, hydraulic conductivity, and permeability of the membrane’s decline lead to the reduced growth. Under saline conditions, Na^+^ replaces Ca^2+^ in the cell walls and interferes in the cells' normal function, leading to ionic imbalances and impacting the growth and yield of plant^47^. Ca^2+^ as a secondary messenger plays pivotal roles in cell actions and selenium holds a dominant role in Ca^2+^ homeostasis inside cells. Furthermore, Ca^2+^ has a role in regulating plant growth, development, and cell membrane integrity^48^. Under salinity conditions with the increased ROS generation, cells face chaos in molecular signaling (variations in inside cell Ca content). Hence, cells balance the situation by translocating Ca^2+^ from the endoplasmic reticulum, Golgi vesicles, or vacuoles towards the cell walls. Increased stress and the continued calcium replacement process cause cell damage and death. Seemingly, Se foliar spray controls the complications by reducing ROS levels and even regulating Ca^2+^ homeostasis to help the plant survive under stressful conditions^47,48^. The reduced Mg^2+^ absorption and the declined chlorophylls and carotenoids biosynthesis, as well as chloroplasts breakdown, led to the diminished growth potential and yield of plants under salinity^49^. Possibly, Se application under saline conditions improves the absorption of the nutrients and, the equilibrium in the nutrients ratios improves plant growth potential and survival rate^50^. In melon crops, selenium nano-particle increased salinity tolerance by activating antioxidant enzymes, proline content, and the declined content of MDA and H_2_O_2_^51^. Zinc is an essential nutrient for the biosynthesis of phenolics, smoothening the ROS effects and maintaining membrane integrity^52^. Fe foliar application, in contrast, reduces the absorption of Zn by the strong competition results in the diminished Zn absorption^53^. Salinity stress harmed the nitrogen content of tomatoes. However, spraying the plant with iron-chelate increased nitrogen content^54^. Salinity stress in plant tissues disturbs the balance between nutrients and interferes with nitrogen metabolism. The use of nano Fe_3_O_4_ in *Moringa* under salinity conditions improved N, P, K^+^, Mg^2+^, Fe, and Zn and, contrarily declined Na^+^ and Cl^-^ content^14^. The nano-particles application under salinity led to the ions equilibrium, reduced Na^+^ toxicity, elevated K^+^ uptake, intensified antioxidant defense, and improved the stomatal conductance and hence, enhanced survival rate under stressful condition^55^.

The low diameter of nano-Fe particles goes to the feasible Fe absorption especially under stressful conditions, helps in the nutrients balance and, via the equilibrium of other nutrients, improves the nutrients use efficiency and photosynthesis potential and, finally, the growth of plants.

## Methods

The homogeneous one-year-old rooted-cuttings of *Vitis vinifera* L. cv. Sultana were provided by the nursery of the Horticultural Science Department, University of Maragheh, Iran, following the relevant institutional and national guidelines and legislation. They were cultured in 5-L pots containing soil with loamy sand texture (48% sand, 25% clay, 26% silt) during 2019–2020 in Azarbaijan Shahid Madani University. Soil characteristics were; pH about 7.5, the electrical conductivity of 1.95 dS/m, and organic matter of 0.9%. Graphene oxide (0, 50, and 100 g kg^−1^ of soil) was mixed with the pot’s soil. For the initial growth and the adaptation of grapevines to greenhouse conditions (16 and 8 h of light and darkness, 30:25 °C day and night temperature); the plants were irrigated with tap water for 30 days. Irrigation was regularly done once a week with 350 ml of water. The drained water was returned to the pots again. This irrigation regime was followed during the salinity stress time-course. One month later, when the plants had 5–6 leaves, the salinity stress treatments (0, 50, and 100 mM NaCl) were applied. To prevent the shock of sudden salt consumption, salinity stress was applied periodically with an initial concentration of 25 mM followed by gradual 4 days intervals up to 100 mM. The first foliar application (the concentrations of 0 and 3 mg L^−1^ of Se and magnetic Fe nanoparticles) was performed simultaneously with salinity stress (6–8 leaves), and the second foliar application was applied 15 days later. One month after the last foliar application, samplings were done to study the desired traits. The experiment was composed of 9 treatments, three replications and 81 pots. Three pots were considered as an experimental unit.

### Preparation of graphene oxide

5 g graphite powder and 5 g sodium nitrate were weighed. They were placed in an ice-bath while being stirred and, 230 ml sulfuric acid was gradually added. Then, 30 g potassium permanganate was added. The solution was left at room temperature overnight. The next day, the solution was heated with 500 ml distilled water to 100 °C, and the reaction was allowed to proceed. The resulting graphene oxide was washed in ethanol for 24 h and left at 65 °C for several hours to dry.

### SEM images

**Figure 7:**
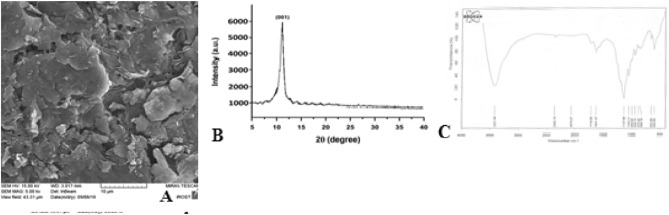
SEM (**A**), XRD spectra (**B**), and FT-IR spectra (**C**) of graphene oxide nanoparticles.

Scanning electron microscopy was employed to investigate the morphological features of the synthesized nanoparticles. Figure 7A shows the SEM image of magnetic graphene nanoparticles. This image reveals that the graphene oxide plates were well-synthesized.

### XRD spectrum

The XRD spectra of the synthesized nanoparticles are shown in Fig. 7B. The peak observed at 2ϴ (equals 11°) confirms the synthesis of graphene oxide.

### FT-IR spectrum

The infrared spectroscopy of the Fourier transform is often used to determine the nature and confirm the presence of functional groups in the structure. Figure 7C shows the FT-IR spectrum of graphene oxide nanoparticles in the 500–4000 cm^−1^ range. In the region 3421 cm^−1^, a broad peak with relatively high intensity was seen; which was related to the tensile vibrations of O–H. A weak peak was observed in the region 2849–2926 cm^−1^, related to C–H tensile vibrations. In the region 1719 cm^−1^, a weak peak related to the tensile vibrations of the carbonyl group (C=O); and in the region 1631 cm^−1^, the peak related to the tensile vibrations of C=C were found. The C–O flexural and tensile vibrations were seen in 1008–1137 cm^−1^. It is noteworthy that the weak peak observed in the region 2363 cm^−1^ was related to ambient CO_2_^56^.

### Synthesis of Fe_3_O_4_ nanoparticles

First, the silicone oil was poured into the crystallizer dish and placed on the heater. The heater degree was set at 100 °C and a thermometer was put inside it to stabilize the bath temperature at 80 °C. A single-mouth balloon containing 40 ml distilled water was inserted into the ultrasonic and 4.83 g hexahedron chloride and 3.34 g heptahydrate Fe sulfate were added. Then, the balloon was placed in the silicone oil using a base and clamp and stirred by a magnet as much as possible. Once the salts were dissolved entirely, 12 ml concentrated ammonia was added to the solutions immediately. Adding ammonia changed the color of the solution immediately to black. The balloon was closed with a cap or parafilm and stirred for up to two hours in this condition. The system was checked from time to time to control the temperature and agitation. Then, the balloon was allowed to reach the ambient temperature and the contents of the balloon were separated from the reaction solution. Later, it was washed three times using a 1:1 solution of ethanol: water to remove the remaining reactants and was dried in an oven at 80 °C.

### Features of synthesized Fe_3_O_4_ magnetic nanoparticles

XRD and FTIR techniques were used to evaluate the synthesis accuracy and the features of Fe_3_O_4_ magnetic nanoparticles.

### XRD spectrum

**Figure 8:**
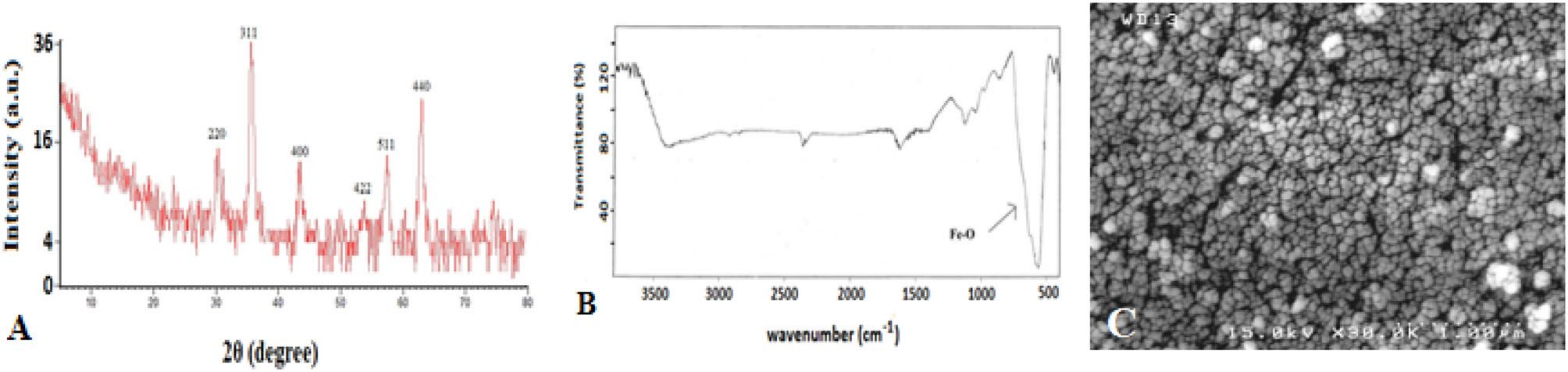
XRD spectrum (**A**), FT-IR spectrum (**B**), and FE-SEM (**C**) of Fe_3_O_4_ nanoparticles.

The XRD spectra of the synthesized nanoparticles are shown in Fig. 8A. The peaks formed in 2θs equal to 30.007°, 53.782°, 43.239°, 35.601°, 30.007°, and 63.058° confirm the synthesis of Fe nanoparticles.

### FT-IR spectrum

The infrared spectroscopy of the Fourier transform is often used to determine the nature and confirm the presence of functional groups in the structure. Figure 8B shows the FT-IR spectrum of Fe_3_O_4_ nanoparticles in the range of 500–4000 cm^−1^. In this figure, a peak is seen around 570 cm^−1^, related to the tensile vibrations of Fe–O, which indicates that Fe–O particles were well synthesized.

### FE-SEM images

Scanning electron microscopy was used to investigate the morphological features of the synthesized nanoparticles. Figure 8C shows the FE-SEM image of Fe_3_O_4_ nanoparticles.

### Plant dry weight

The plants were harvested when they had 15–17 leaves. A precise digital scale assayed the fresh weight of above-ground parts. The dry weight of chopped (5–7 cm in size) plant materials was measured after drying at 25 °C until constant weight.

### Sampling for MDA, hydrogen peroxide, and proline

During the 15–17 leaf stage, leaf samples were taken from the 10–12 nodes, wrapped in aluminum foil, frozen in liquid nitrogen, and immediately were transferred to the lab.

### Malondialdehyde content

0.2 g of frozen grape leaf samples were homogenized with 5 ml, 1% volumetric TCA (volume/ weight), and centrifuged at 12,000 g for 15 min. Then, 1 ml of supernatant was mixed with 4 ml TCA 20% + TBA 0.5% and heated for 30 min. The enzyme activity was then rapidly stopped by placing the samples on ice. The absorbance of the samples was read by spectrophotometer (T80 + , China) at 532 and 600 nm^57^.

### Hydrogen peroxide content

0.2 g of frozen grape leaf samples was homogenized with 5 ml 1% volume TCA (volume/weight). The homogenous solution was centrifuged for 12 min at 12,000*g*. Then, the hydrogen peroxide content was measured based on the method of Amaranathareddy et al.^58^. The absorbance of the samples was read at 390 nm. Standard curves were established with the different concentrations of hydrogen peroxide.

### Superoxide dismutase activity

SOD activity was determined by measuring the inhibition of light reduction of nitroblutetrazolium at a wavelength of 560 nm. Doing this, 50 ml of 50 mM potassium phosphate buffer, pH: 7.5, was used. Then, 75 μM nitroblutetrazolium, 13 μM methionine, 0.1 μM EDTA solution, and 4 μM riboflavin were added to the buffer and the solution was stored in a dark place^59^.

### Catalase activity

0.5 g of grape leaf samples was homogenized with 0.1 M cold potassium phosphate buffer (pH: 7.5) with 0.5 mM EDTA based on the method of Dezar et al.^60^. From the resulting supernatant, 0.05 ml was added to 1.5 ml of 0.1 mM phosphate buffer (pH: 7) and 1.45 ml double distilled water. The reaction was started by adding 0.5 ml of 75 mM hydrogen peroxide and a decrease in adsorption was recorded at 240 nm for 1 min.

### Proline content

Proline content was assayed according to the method of acid-ninhydrin and toluene at 520 nm, as described by Fedina et al.^61^. The proline content was computed using a standard curve of proline, and the results were expressed as micrograms of proline per gram of plant fresh weight.

### Phenolics and flavonoids content

Total phenolics and flavonoids were determined via Kim et al.^62^ using Folin-Ciocalteu reagent. Gallic acid was employed as the standard.

### Total chlorophyll content

Chlorophylls content was determined in acetone extract according to Dere et al.^63^. The absorbance was read spectrophotometrically at 663 and 645 nm.

### Total soluble solids content (°Brix)

The filtered leaf extracted juice was used to determine TSS by a digital refractometer (Erma, Tokyo, Japan).

### Leaves mineral elements content

The flame photometric method (Corning, 410, England) was employed to measure the amount of sodium and potassium. The atomic absorption spectrometer (Corning, 410, England) measured Zn, Fe, Ca, P, Mg, and Mn content (Corning, 410, England). Kjeldahl methods quantified N content. The graphite furnace quantified Se content.

### Experimental design and data analysis

The present experiment was performed as a factorial based on a completely randomized design with three replications. MSTATC (ver. 2.1, Michigan University), Minitab (ver. 17), and R Software (ver. 3.6.3) were used to the ANOVA, cluster, biplot, and correlation analysis of data, respectively. The means were compared using Duncan’s multiple range tests at 5 and 1% probability levels.

## Conclusion

Salinity stress impacted the growth potential, nutrient content, and some physiological responses of the grapevine. However, nano-graphene oxide and foliar treatments improved aerial parts growth and plant height. Under salinity, graphene oxide application improved MDA, TSS, and chlorophyll a content. Meanwhile, with 100 mM salinity, grapheme oxide use had no positive effect on MDA content. Overall, under salinity; graphene oxide, Se, and nano-Fe treatment improved antioxidant enzymes activity, osmolytes, and the mineral nutrients balance in favor of growth potential. Graphene oxide, Se, and nano-Fe treatments improved the plant responses up to the salinity of 50 mM. Eventually, it seems that the programmed application of nano-carbons like graphene oxide and the nano-nutrients would be a promising alternative in coping with the salinity adverse effects on plants by ameliorating the salinity depression via the enhanced growth potential, antioxidants metabolism, and the improved nutrients availability.
